# Anxiety during COVID-19 among School Going Adolescents of Six Private Schools in Kathmandu Valley: A Descriptive Cross-sectional Study

**DOI:** 10.31729/jnma.5703

**Published:** 2021-03-31

**Authors:** Anwesh Bhatta, Rishi Kesh Kafley, Arabindra Yadav, Rajan Phuyal, Vijaya Kumar Chikanbanjar

**Affiliations:** 1 Department of Pediatrics, Kathmandu Medical College Teaching Hopsital, Sinamangal, Kathmandu, Nepal

**Keywords:** *adolescent*, *anxiety*, *COVID-19*, *pandemics*

## Abstract

**Introduction:**

Psychological health problems are common in adolescent and young adults. The psychological well-being is greatly influenced by stressful environment and the coping mechanism of an individual. The 2019 Coronavirus disease has caused unprecedented morbidity and mortality worldwide owing to its high infectivity and mortality. In addition to these physical manifestations, psychological impact has also been substantial. This study is a descriptive cross-sectional study done to find out the prevalence of anxiety disorder and its severity during the COVID-19 pandemic in school going adolescents of Kathmandu valley.

**Methods:**

A descriptive cross-sectional study was carried out among adolescents from August 15 to September 30, 2020. Two private schools from each district in the Kathmandu valley i.e. Kathmandu, Lalitpur and Bhaktapur were included in the study using convenient sampling. Ethical clearance was obtained from Institutional Review Committee (reference no: 1208202007). All data were inserted on Microsoft Excel 2016 and analysed using Statistical Package for the Social Sciences version 20. Point estimate at 95% Confidence Interval was calculated along with frequency and proportion for binary data.

**Results:**

Among 358 students, 165 (46.1%) at 95% Confidence Interval (40.8-51.2) were found to have anxiety. Out of these patients, 115 (69.7%) had mild, 39 (23.6%) had moderate and 11 (6.7%) had severe anxiety. The mean age was 16.17±1.57 years.

**Conclusions:**

This study shows that a remarkably higher number of children had symptoms of anxiety disorder. Majority of the children with anxiety had mild form. The study further highlights the need of emotional support to adolescent children during the current ongoing pandemic.

## INTRODUCTION

Mental health problems are prevalent in adolescent age group. Mental health problems account for 16% of global disease burden and injury in the age group 10-19 years. Globally, anxiety is the ninth leading cause of illness and disability for adolescents aged 15-19 years and sixth for those aged 10-14 years.^1^

Stressful life events break down the delicate defence mechanism leading to subtle clinical manifestations being evident. During the coronavirus disease (COVID-19), numerous studies have highlighted the psychological impact of the pandemic and have highlighted the increased risk on the mental well-being of adolescents.^2-4^ This unprecedented, unpredictable disease has been causing universal awareness along with considerable anxiety and distress which according to the World Health Organization (WHO) is a natural psychological response.^5^

This study was conducted among adolescent school going children to find out the prevalence of generalized anxiety disorder and its severity using the Generalized Anxiety Disorder (GAD-7) tool.

## METHODS

This is a descriptive cross-sectional study conducted among adolescents attending private schools in the Kathmandu valley during the month of August 15^th^ to September 30^th^, 2020 after obtaining ethical clearance from Institution Review Committee (IRC) of Kathmandu Medical College Teaching Hospital (Reference No. 1208202007). Two private schools from each district in the Kathmandu valley i.e. Kathmandu, Lalitpur and Bhaktapur were included in the study using convenient sampling. E-mail address of all the students studying in these schools from class 8-12 were collected and an online form was sent to all these students. The students were informed about the study and those students who voluntarily filled up the online form were regarded as having consented to participate in the study. All adolescents studying in grade 8-12 in these private schools of Kathmandu valley who responded to the online form were included in the study. The possibility of respondent bias with only the students interested in the topic filling up the online form was kept in mind. To minimise this, repeated reminder emails up to three times were sent to students who did not respond the first time to reduce the chance of this bias.

The reported incidence of anxiety during COVID-19 in adolescents has been 26%.^6^ Convenience sampling was done and the sample size was calculated using the formula,

$$\begin{array}{ll} \text{n} & ={\text{Z}}^{\text{2}}\times \text{p}\times \text{q}/{\text{e}}^{\text{2}}\\ & ={\left(1.96\right)}^{2}\times (0.26)\times (1-0.26)/{\left(0.05\right)}^{\text{2}}\\ & =296 \end{array}$$

Where,

n = required sample size,
Z = 1.96 at 95% Confidence Interval
p = prevalence, 26%^6^
q = 1-p
e = margin of error, 5%

After adding 10%, non-response rate the sample size was calculated to be 326. However, a total of 592 subjects were sent the questionnaire for the study as an online form. Of these, 358 students filled up the form and returned.

All students were requested to fill an online questionnaire form which was sent to them via e-mail. The questionnaire comprised of two parts. The first part of the questionnaire comprised of demographic data including gender and age of the subject while the second part of the questionnaire was the GAD-7 tool. The seven-itemed GAD-7 tool is a widely used tool for screening anxiety disorder.^7^

All the data were subsequently filled up in Microsoft Excel 2016. Statistical analysis was done in Statistical Package for the Social Sciences (SPSS) version 20. Descriptive analysis including frequency, mean, median and standard deviation were calculated on the SPSS 20. Point estimate at 95% Confidence Interval was calculated along with frequency and proportion for binary data.

## RESULTS

A total of 358 students were enrolled in the study. Generalized anxiety of some form was reported in 165 (46%) at 95% CI (40.8-51.2) of the participants. Of the 165 patients reported to have some form of anxiety, 115 (69.7%) had mild anxiety, followed by moderate anxiety in 39 (23.6%) and severe anxiety in 11 (6.7%) (Table 1). The mean age of the students was 16.17±1.57 years. Among the enrolled subjects, majority were female with 184 (51.4%).

**Table 1:** Frequency of anxiety based on its severity.

Severity of anxiety	n (%)
Mild anxiety	115 (69.7)
Moderate anxiety	39 (23.6)
Severe anxiety	11 (6.7)
Total	165 (100)

Girls 96 (52%) tended to report more anxiety symptoms as compared to boys 69 (39%) and that was true in all the severity of anxiety (Table 2).

**Table 2:** Gender based distribution of anxiety.

Gender	No anxiety n (%)	Mild anxiety n (%)	Moderate anxiety n (%)	Severe anxiety n (%)	Total n (%)
Male	105 (54.4)	49 (42.61)	16 (41.02)	4 (36.3)	174 (48.6)
Female	88 (45.6)	66 (57.4)	23 (58.9)	7 (63.6)	184 (51.4)
**Total**	**193**	**115**	**39**	**11**	**358**

## DISCUSSION

Psychological health problems are common in adolescent and young adults.^3^ The psychological wellbeing is greatly influenced by stressful environment and the coping mechanism of an individual.^6^ The 2019 Coronavirus disease (COVID-19) has caused unprecedented morbidity and mortality worldwide owing to its high infectivity and mortality. In addition to these physical manifestations, psychological impact has also been substantial.^8^

This study revealed that 46% of the participating students had some form of anxiety. A majority of them had mild form of anxiety followed by moderate and severe form of anxiety. A similar study done among college students in China revealed 24.9% of the respondents had some form of anxiety with majority of these students having mild form of anxiety comprising around 85.5%.^4^ Another study done in the epicentre of COVID 19 pandemic at Wuhan revealed that 27% of all 7772 participating adolescent students had anxiety.^9^ Similarly in another study done in China, anxiety was present in 37.4% of 8079 high school attending adolescents.^10^ The higher prevalence of anxiety in our study might reflect a smaller sample size as compared to the other studies.

Our study revealed that females had more anxiety symptoms as compared to males. Females outnumbered males in cases with anxiety symptoms in similar other studies too.^4,9,10^

Our study has multiple limitations. Measurement of anxiety in our study was done using self-reported questionnaire which could introduce potential biases. Secondly, as this was an online study, the study was not representative of students who did not have access to the internet. Lastly, the limited sample size and the convenient sampling technique could not make the results representative of the entire population.

## CONCLUSIONS

The purpose of the study was to find the prevalence of anxiety during the COVID 19 pandemic. The prevalence of anxiety was found to be higher than during non-COVID times. However, the government and the private sector has not given due attention to the psychological impact of the pandemic in this vulnerable adolescent age group. Due attention and intervention could potentially decrease the possible untoward incidents in the future.
